# Cost-Effectiveness of a Quality of Life Predictor to Guide Psychosocial Support in Breast Cancer

**DOI:** 10.3390/cancers18030439

**Published:** 2026-01-29

**Authors:** Tuukka Hakkarainen, Ira Haavisto, Mikko Nuutinen, Yrjänä Hynninen, Paula Poikonen-Saksela, Johanna Mattson, Haridimos Kondylak, Eleni Kolokotroni, Ketti Mazzocco, Berta Sousa, Isabel Manica, Ruth Pat-Horenczyk, Riikka-Leena Leskelä

**Affiliations:** 1 Nordic Healthcare Group, 02150 Helsinki, Finland; 2 Comprehensive Cancer Center, University of Eastern Finland, 70211 Kuopio, Finland; 3 Department of Oncology, Comprehensive Cancer Center, Helsinki University Hospital, University of Helsinki, 00290 Helsinki, Finland; 4 Institute of Computer Science (ICS) Foundation for Research and Technology—Hellas (FORTH), 700 13 Heraklion, Greece; 5 In Silico Oncology and In Silico Medicine Group, Institute of Communication and Computer Systems, School of Electrical and Computer Engineering, National Technical University of Athens, 157 80 Zografos, Greece; 6 Applied Research Division for Cognitive and Psychological Science, European Institute of Oncology, IRCCS, 20141 Milan, Italy; ketti.mazzocco@unimi.it; 7 Breast Unit, Champalimaud Clinical Centre, Champalimaud Foundation, 1400-038 Lisboa, Portugal; 8 Paul Baerwald School of Social Work and Social Welfare, The Hebrew University of Jerusalem, Jerusalem 7612001, Israel

**Keywords:** breast cancer, resilience, psychosocial support, machine learning, clinical decision support, quality of life, cost-effectiveness, decision-analytic modeling, net monetary benefit, healthcare utilization

## Abstract

**Introduction**: Women with breast cancer experience psychological distress, and resilience-strengthening psychosocial support may improve their quality of life (QoL). Identifying those at risk of low QoL is challenging. This study evaluated the cost-effectiveness of a machine learning-based QoL predictor to support clinical decision-making regarding psychosocial support (sample size: 660). **Methods**: A decision tree cost–utility model was developed to compare four decision-making strategies in offering psychosocial support: the clinician alone, the QoL predictor alone, the clinician supported by the predictor, and no prediction with no psychosocial support. QoL after one year was used as a proxy for resilience. Costs, health outcomes, and net monetary benefits (NMBs) were estimated using a one-year time horizon. Incremental cost-effectiveness ratios (ICERs) were calculated and dominance assessed. A societal scenario analysis incorporated productivity losses. A probabilistic sensitivity analysis generated cost-effectiveness acceptability curves. **Results**: Clinicians supported by the QoL predictor produced the highest NMB (EUR 16,349) and the greatest quality-adjusted life year (QALY) gain (0.759), with an ICER of EUR 22,892 compared with the next least costly strategy. Clinician-only prediction and predictor-only approaches were dominated or extendedly dominated. Under the societal perspective, all strategies produced negative NMB values due to productivity losses, but the overall ranking remained unchanged. The probabilistic sensitivity analysis showed that the combined clinician and predictor strategy had a 69% probability of being cost-effective at a willingness to pay threshold of EUR 30,000. **Conclusions**: Combining clinician judgement with the machine learning-based QoL predictor improved the targeting of psychosocial support and was the most cost-effective strategy. Further prospective and comparative studies are needed to confirm its long-term effectiveness and cost-effectiveness in clinical practice.

## Simple Summary

Women diagnosed with breast cancer often experience psychological distress, and some benefit from psychosocial support to help maintain their quality of life. However, identifying which patients are most likely to need this support can be difficult in routine clinical practice. This study examined whether a machine learning tool that predicts future quality of life could help clinicians to make better decisions about offering psychosocial support. We compared four decision-making strategies, including the clinician alone, the machine learning tool alone, and the combination of both. Using health economic modeling based on observational data from women with breast cancer, we estimated the health benefits, healthcare costs, and overall value of each strategy. Our findings suggest that combining clinicians with support from the prediction tool may improve decision-making and help to target psychosocial support to the patients who may benefit most.

## 1. Introduction

Breast cancer is the most common cancer globally and accounts for approximately 30% of all cancers among women in Europe [1]. Despite advancements in cancer treatment leading to improved prognoses, cancer diagnosis is associated with significant psychological distress [2].

Resilience is the capacity to maintain or regain psychological and physical equilibrium when facing substantial stress [3]. In oncology, resilience is conceptualized as a dynamic process of adaptation to cancer-related adversity [4]. Across studies of patients and survivors, higher resilience is associated with better adjustment to cancer, superior quality of life, and fewer symptoms of psychological distress [5,6,7,8,9,10].

Patients with higher resilience report fewer symptoms of anxiety and depression and better physical, emotional, and social functioning, resulting in higher quality of life (QoL) [9]. Cancer patients’ high resilience may buffer against psychological distress and improve the QoL of the patient during the course of the disease [5]. Evidence suggests that high resilience, or its fortification, not only has an immediate impact but also confers benefits up to six years post-cancer diagnosis [2]. A study involving individuals with advanced-stage cancer revealed a direct correlation between high resilience and higher levels of perceived social support, as well as reduced feelings of hopelessness [11].

Interventions aimed to strengthen resilience, including digital support, physical exercise, psychological therapies, and other complementary methods, have demonstrated a positive impact on QoL [12,13,14]. Distinguishing patients who exhibit high resilience and thereby maintain high QoL after breast cancer treatment from those who do not is crucial for both clinical practice and broader societal implications [15,16].

Clinical decision support systems (CDSS) can help clinicians to identify target patients and to determine appropriate interventions [17]. In recent years, clinical decision support systems equipped with machine learning algorithms have seen increasing application in healthcare, aiding clinicians in critical tasks such as diagnosis, subtyping, and evaluating patient responses to treatments [18,19,20,21,22,23]. Machine learning-based digital health applications have the potential to improve decision-making and access to healthcare and enable cost savings [24].

Clinical decision-making is inherently complex and prone to cognitive biases [25]. As discussed by Hakkarainen et al. (2025) [26], the predictive performance of an algorithm alone does not necessarily translate into clinical utility in real-world settings. Therefore, the human component of the decision-making process must be explicitly considered when assessing the cost-effectiveness of predictive tools. In practice, successful implementation will also depend on clinician acceptance, integration into existing workflows, and adequate training, all of which may influence real-world effectiveness.

The quality of life (QoL) predictor is a machine learning-based CDSS [27] that estimates the probability of mental health deterioration in women with breast cancer within 12 months after assessment. It provides both a probabilistic risk estimate and a binary high-versus-low risk classification, serving as a complement to individual, needs-based clinical assessment. The QoL predictor integrates routinely available electronic health record data with patient-reported outcomes to identify those most likely to experience low QoL one year after diagnosis—patients who may benefit most from resilience-strengthening interventions [27,28].

Although previous research has highlighted the importance of supporting patients with low resilience [5,6,7,8,9,10], reliably identifying those at risk in routine clinical practice remains challenging. An algorithmic approach to predicting mental health deterioration could therefore help clinicians to recognize patients who are most likely to benefit from psychosocial support. However, the cost-effectiveness of such algorithm-guided decision-making compared with usual care—where no structured risk assessment tool is used—has not been established. The aim of this study was to conduct an economic evaluation to estimate the cost-effectiveness of the QoL predictor relative to the current standard of care.

## 2. Methods

### 2.1. The Quality of Life Predictor

This economic evaluation was conducted as part of the EU-funded multicenter clinical study BOUNCE (H2020 project, Grant Agreement No. 777167). Patient data were collected from 660 women with histologically confirmed stage I–III breast cancer. The clinical recruitment sites were from four different countries:

1. Helsinki University Hospital Comprehensive Cancer Center (HUS), Finland;
2. Hebrew University in Jerusalem, Israel;
3. Champalimaud Breast Unit (CHAMP), Portugal;
4. The European Institute of Oncology (IEO), Italy.

The study was approved by the European Institute of Oncology, Applied Research Division for Cognitive and Psychological Science (Approval No. R868/18—IEO 916) and by the institutional ethics committees of all participating hospitals. The full study protocol has been published [29].

The QoL predictor was developed using data from the multicenter BOUNCE cohort, as described in detail by Kourou et al. 2023 [27]. The model was trained on psychosocial, clinical, sociodemographic, and lifestyle variables collected at baseline and three months after diagnosis. A balanced random forest approach with cross-validation was used, achieving good predictive performance (AUC up to 0.86 for one-year mental health deterioration) and interpretable outputs through established explanation techniques. A user experiment [28] demonstrated that clinicians’ prediction accuracy improved when supported by the tool, providing the rationale for evaluating its cost-effectiveness.

### 2.2. Quality of Life as an Outcome

QoL can serve as a pragmatic outcome for resilience [15,29], as resilience is a complex and multidimensional construct that can be defined at different levels [30]. In the BOUNCE study, QoL was used as a proxy indicator of resilience, where the QoL predictor predicted whether a patient’s QoL one year after breast cancer diagnosis would be classified as low or high [27,28]. A QoL score of ≤0.75 was used as the threshold for low QoL [28]. Hereafter, mental health deterioration prediction is referred to as “QoL prediction”, reflecting the QoL predictor’s use of QoL as a proxy for resilience.

### 2.3. Decision-Analytic Model

**Figure 1:**
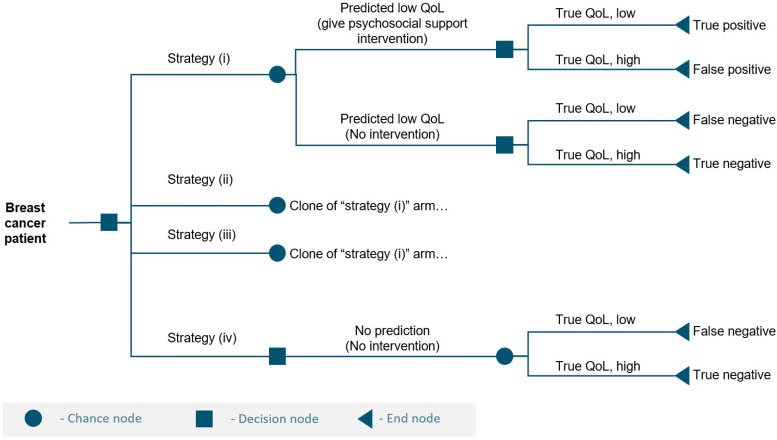
A simplified decision tree representing the four decision-making strategies evaluated in the model. QoL = quality of life. Strategies: (i) clinician prediction, (ii) clinician prediction supported by the QoL predictor, (iii) QoL predictor alone, (iv) no QoL prediction and no psychosocial support.

We developed a decision-analytic cost–utility model to evaluate the cost-effectiveness of using the QoL predictor to guide psychosocial support decision-making among women with breast cancer. The model structure was based on a decision tree framework representing four distinct clinical decision-making strategies (Figure 1). Three of the strategies incorporated QoL prediction, as evaluated in the user experiment by Nuutinen et al. 2023 [28].

(i) Clinician-only QoL prediction: A clinician independently predicts whether a patient will have low or high QoL one year after diagnosis using the available clinical and psychosocial information. Psychosocial support is provided to patients predicted to have low QoL.

(ii) Clinician prediction supported by the QoL predictor: A clinician makes an initial QoL prediction and then reviews the QoL predictor’s output before making a final decision. Psychosocial support is provided to patients classified as having low QoL after this combined decision process.

(iii) QoL predictor-only prediction: The machine learning-based QoL predictor autonomously determines whether a patient will have low or high QoL at one year based on the same clinical and psychosocial inputs. Psychosocial support is provided to patients predicted to have low QoL.

In all prediction-based strategies, psychosocial support was provided only to patients predicted to have low QoL. A fourth strategy represented a non-prediction approach, corresponding to current standard practice when structured QoL prediction decision-making processes are not used.

(iv) No prediction and no psychosocial support: No QoL prediction is performed, and psychosocial support is not provided.

### 2.4. Psychosocial Support Intervention

The effects of psychosocial support for this cost and benefit analysis were derived from the randomized controlled trial by Arving et al. (2007) [30], which evaluated individual psychosocial support (IPS) for women with primary breast cancer. The IPS intervention, grounded in cognitive behavioral therapy, comprised individual sessions (mean 4.5; range 0–23) of 45–60 min over approximately six months. In this model, IPS was treated as a resilience-strengthening intervention, with two decision options: IPS or no intervention. The benefits were modeled as improvements in QoL. Only patients with true low QoL were assumed to benefit from the IPS.

### 2.5. Clinical Utility

In this study, clinical utility was derived from the user experiment conducted by Nuutinen et al. (2023) [28], which provided empirically derived estimates of combined human–algorithm decision performance; these were translated into sensitivity and specificity factors for use in the economic model. These parameters, summarized in Table 1, reflect the potential real-world clinical utility of the QoL predictor in clinical practice.

### 2.6. Utility

Utility values were derived from the BOUNCE dataset [29], where the study used the EORTC QLQ-C30 Global Health Status/QoL scale [31] to assess the quality of life of women with breast cancer. The questionnaire was administered at baseline and after 12 months of follow-up. Responses were converted to a utility scale ranging from 0 (equivalent to death) to 1 (perfect health). At baseline, the mean utility score was 0.74, increasing slightly to 0.76 at one year.

### 2.7. Costs and Healthcare Resource Use

Data on healthcare service use among breast cancer patients were obtained from the BOUNCE dataset [29]. Information on sick leave days was derived from a Finnish national register study by Leskelä et al. 2023 [32]. Mean annual values were calculated separately for patients with high and low QoL one year after diagnosis. To estimate the effect of IPS on healthcare use and productivity losses, the association between QoL and the number of healthcare visits and sick leave days over the one-year period was modeled using linear regression analysis.

The unit cost of IPS was derived from national average healthcare service prices in Finland [33] (Table 2). The costs for each prediction method were calculated using the human capital method, factoring in the average time that a clinician spends in making the prediction and intervention decision. The per-patient cost of using the predictor was determined based on assessments from clinical partners.

Unit costs for healthcare visits were based on the mean total cost of an elective oncology specialist visit in Finland [34]. The cost of a sick leave day was estimated using the human capital approach based on average labor costs per workday for Finnish employers [35]. All costs were converted to 2024 EUR using the producer price index for services of Statistics Finland [36].

### 2.8. Model Assumptions

The prior probability of low QoL one-year after breast cancer diagnosis was estimated from the BOUNCE population data [29]. These prior probabilities were updated using Bayesian inference based on observed prediction results [37]. Patients’ QoL was represented as a binary outcome (low vs. high) one year after diagnosis. It was assumed that true QoL remained constant during the decision-making process—only the belief about a patient’s QoL changed. Predictions were considered conditionally independent, as the joint distribution of results from different prediction sources was unavailable.

### 2.9. Analysis

The analysis was conducted from a Finnish third-payer perspective with a one-year time horizon, corresponding to the primary follow-up period of the BOUNCE study. The target population was aligned with the inclusion criteria applied during the development of the QoL predictor and comprised females aged 40–70 years at diagnosis with histologically confirmed invasive breast cancer (stage I–III), encompassing early or locally advanced but operable disease, who received surgery as part of local treatment and systemic therapy regardless of the treatment type.

Expected per-patient costs and QALYs were estimated for each of the four decision-making strategies. Because of the one-year analytic horizon, no discounting was applied to either costs or health outcomes. A willingness to pay (WTP) threshold of EUR 30,000 per QALY gained was used in the base case analysis. The primary outcomes were the incremental cost-effectiveness ratio (ICER), expressed as the cost per QALY gained, and the mean net monetary benefit (NMB) per patient.

The incremental cost-effectiveness analysis followed standard procedures. Strategies were first ordered by increasing mean cost and then compared sequentially. Strict dominance (higher costs combined with lower QALYs than another strategy) and extended dominance (an ICER exceeding that of a more effective strategy) were assessed. The cost-effectiveness frontier was constructed by retaining only the non-dominated strategies and plotting their mean costs and QALYs on the cost-effectiveness plane, with QALYs on the x-axis and costs on the y-axis.

All analyses were conducted using base case parameter values derived from the BOUNCE dataset and published literature. The model was implemented in Microsoft Excel and validated internally for structural and face validity before running simulations. This economic evaluation adhered to the Consolidated Health Economic Evaluation Reporting Standards (CHEERS 2022) checklist [38] to ensure methodological transparency and reproducibility. No separate health economic analysis plan was preregistered.

### 2.10. Scenario and Sensitivity Analyses

A scenario analysis adopting a societal perspective was performed to evaluate the impact of including productivity losses arising from sick leave on the cost-effectiveness results.

To investigate the uncertainty surrounding the parameters in the model, we conducted a probabilistic sensitivity analysis. This involved running 10,000 Monte Carlo simulations for each WTP threshold, ranging from EUR 1 to EUR 140,000. In each iteration, model parameters were randomly sampled from predefined probability distributions to reflect parameter uncertainty. The distributions for the random sampling were as follows: costs were assigned gamma distributions, utilities followed beta distributions, and regression coefficients were sampled from normal distributions.

The probabilistic sensitivity analysis results were used to construct cost-effectiveness acceptability curves, illustrating the probability of each strategy being cost-effective at varying WTP thresholds. This approach provided the robust quantification of uncertainty and tested the stability of model outcomes under multiple scenarios.

## 3. Results

### 3.1. Base Case Results

The strategy with the highest NMB was clinician prediction supported by the QoL predictor (Strategy ii) at EUR 16,349 per patient. This strategy also generated the highest health benefit, with 0.759 QALYs, while maintaining the second-lowest overall cost (EUR 6414). The clinician-only strategy (Strategy i) and the predictor-alone strategy (Strategy iii) each produced an NMB of EUR 16,327, although their cost and QALY results differed slightly. Clinician prediction alone resulted in 0.758 QALYs at a cost of EUR 6420, whereas the predictor alone resulted in 0.756 QALYs at a lower cost of EUR 6354. The strategy with no QoL prediction and no psychosocial support (Strategy iv) resulted in the lowest QALYs (0.745) and the lowest cost (EUR 6104), resulting in the lowest NMB of EUR 16,252.

Table 3 presents the results of the incremental cost-effectiveness analysis, in which the strategies are ordered by increasing cost and compared sequentially. Strategy iv (no QoL prediction and no psychosocial support) served as the reference as it was the least costly option. Relative to Strategy iv, all three prediction strategies (Strategies i–iii) generated additional QALYs and higher costs. Strategy i (clinician-only prediction) increased the costs by EUR 316 and produced an incremental gain of 0.013 QALYs, resulting in an ICER of EUR 24,255 per QALY gained. Strategy ii (clinician prediction with the QoL predictor) produced an additional incremental gain in QALYs of 0.014 at an incremental cost of EUR 310, resulting in the lowest ICER among the strategies (EUR 22,892 per QALY). Strategy iii (predictor alone) generated 0.011 additional QALYs at EUR 250 of additional cost, resulting in an ICER of EUR 23,056 per QALY.

When examining the cost-effectiveness frontier (Table 3), Strategy i was dominated by Strategy ii, as it produced fewer QALYs at a higher cost. Similarly, Strategy iii was extendedly dominated, as its ICER exceeded that of the more effective Strategy ii. As a result, only Strategy ii remained on the cost-effectiveness frontier, providing the highest health gain per euro spent among the strategies.

### 3.2. Sensitivity Analysis Results

In the scenario analysis, a societal perspective was adopted, where productivity losses due to sick leave were incorporated into the model. This resulted in all strategies generating a negative NMB. Despite the change in the absolute NMB values, the relative ranking of the strategies remained consistent with the base case results. Clinician prediction supported by the QoL predictor (Strategy ii) continued to result in the highest NMB (EUR −22,527), followed by clinician-only prediction (Strategy i; EUR −22,595). The predictor-alone strategy (Strategy iii) resulted in an NMB of EUR −22,790, and the no prediction and no psychosocial support strategy (Strategy iv) produced again the lowest NMB (EUR −23,820). All three prediction strategies produced higher QALYs and lower costs than Strategy iv under the societal perspective.

**Figure 2:**
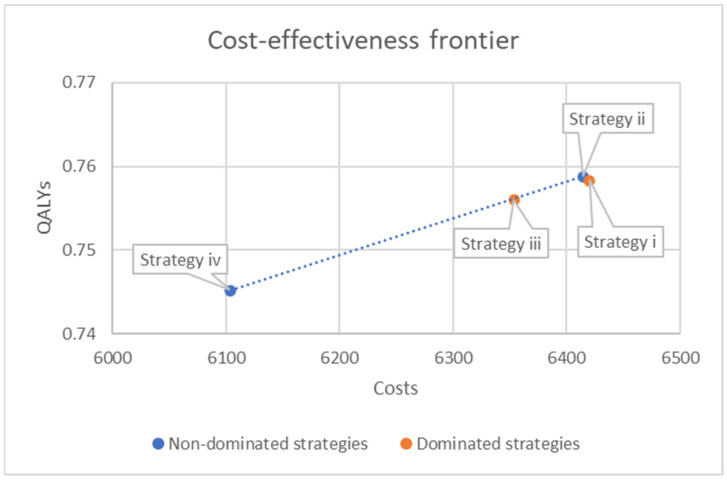
Cost-effectiveness frontier for the four decision-making strategies. QALY = quality-adjusted life year, QoL = quality of life. Strategies: (i) clinician prediction, (ii) clinician prediction supported by the QoL predictor, (iii) QoL predictor alone, (iv) no QoL prediction and no psychosocial support.

**Figure 3:**
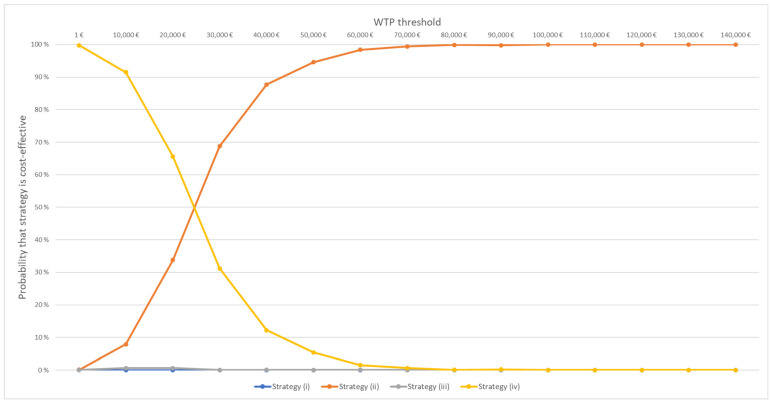
Acceptability curves based on net monetary benefit across 10,000 Monte Carlo simulations. Curves indicate the probability that each strategy yields the highest expected net monetary benefit at varying willingness to pay thresholds. QoL = quality of life. Strategies: (i) clinician prediction, (ii) clinician prediction supported by the QoL predictor, (iii) QoL predictor alone, (iv) no QoL prediction and no psychosocial support.

The results of the probabilistic sensitivity analysis are illustrated in Figure 2 and Figure 3 with cost-effectiveness acceptability, curves which demonstrate the proportion of simulations where each strategy yielded the highest NMB across varying WTP thresholds. Under the assumption of parameter independence, this proportion is interpretable as the probability of each strategy being deemed the optimal strategy at various WTP thresholds. For instance, at the established base case WTP threshold of EUR 30,000, Strategy ii showed a 69% probability of being the strategy with the highest NMB. Strategy iv showed a 31% probability of being the most optimal strategy under the WTP threshold of EUR 30,000, leaving other strategies at 0%. Strategy iv had the highest probability of being the optimal choice until a WTP threshold of EUR 20,000. After this, Strategy ii had the highest probability of being the optimal choice, with the probability increasing as the WTP threshold increased.

## 4. Discussion

This study evaluated the cost-effectiveness of alternative strategies for guiding resilience-strengthening psychosocial support in women with breast cancer. Consistent with the BOUNCE study design [29], QoL was used as a proxy for resilience, with the QoL predictor estimating the likelihood of low versus high QoL one year after diagnosis. Across all base case comparisons, clinician prediction supported by the QoL predictor produced the highest net monetary benefit and the largest QALY gain, strengthening previous findings [17,24] that CDSS can help clinicians to identify target patients for appropriate interventions. The strategy of clinician prediction supported by the QoL predictor also remained the preferred option in the societal scenario analysis and showed the highest probability of being cost-effective at a willingness to pay threshold of EUR 30,000 per QALY.

Including productivity losses in the analysis resulted in negative NMB values for all strategies, indicating that productivity costs outweigh the monetary value of health gains over one year. Negative NMB values in this context do not imply clinical harm but reflect the high economic burden associated with sick leave in the early period following diagnosis and treatment. Despite the change in absolute values, the relative ranking of the strategies remained consistent with the base case, with clinician QoL prediction supported by the QoL predictor resulting in the highest NMB. All three prediction strategies produced higher QALYs and lower costs than the no-prediction strategy (Strategy iv), highlighting the potential societal value of more targeted psychosocial support.

The probabilistic sensitivity analysis showed that clinician QoL prediction supported by the QoL predictor (Strategy ii) had the highest probability of being cost-effective at a WTP threshold of EUR 30,000 per QALY. At lower thresholds, the no-prediction strategy (Strategy iv) was more often the optimal strategy. For countries that do not have an officially defined WTP threshold for QALYs gained with health technologies, evaluating cost-effectiveness across a range of WTP thresholds is particularly important.

Our results support the findings of Seiler et al. 2019 [16], who suggested that improving resilience through targeted interventions could be effective. By using QoL as a proxy for resilience, in line with the previous literature [5,6,7,8,9,10], our study supports the link between higher resilience and better adjustment and quality of life in breast cancer patients. The study also demonstrates the potential value of prediction-based approaches in improving the targeting of resilience-strengthening interventions. The more precise identification of patients at risk of low QoL may reduce unnecessary healthcare use and sick leave. To assess cost-effectiveness in other healthcare systems, the model would need to be adapted to the local context—at a minimum, by updating the system-specific cost inputs.

A common limitation of cost-effectiveness analyses of clinical decision support systems is the assumption of perfect accuracy and compliance [39]. An important strength of our study is that the accuracy, specificity, sensitivity, and compliance parameters were derived from a user experiment [28], providing a more realistic representation of how decisions are made with and without the QoL predictor in clinical practice. Another strength is the use of comprehensive, prospectively collected data from the BOUNCE study [29], which included detailed information on QoL, healthcare service use, and sick leave. Lastly, to account for parameter uncertainty, wide confidence intervals were incorporated into the probabilistic sensitivity analysis.

This study is not without limitations. A key limitation is that the BOUNCE dataset is observational rather than interventional. The analysis relied on estimated associations between psychosocial support and QoL, healthcare use, and sick leave, rather than on effects derived from a head-to-head comparison of intervention versus no intervention. Consequently, the model provides indicative rather than definitive estimates of cost-effectiveness for decision-making strategies and for the targeted psychosocial support. In addition, QoL was modeled as a dichotomous outcome, and the benefits of psychosocial support were conservatively assumed to apply only to patients with truly low QoL, to align the economic model with the clinician–algorithm user experiment by Nuutinen et al. 2023 [28] from which the sensitivity and specificity estimates were derived (Table 1)—an approach that may underestimate real-world effects. Lastly, our analysis used a one-year time horizon, which may have led to the underestimation of potential longer-term benefits of psychosocial support and increased resilience. The static decision tree structure may also fail to capture dynamic feedback processes, such as evolving symptom trajectories, changes in care needs, or repeated decision points. More sophisticated modeling approaches, such as Markov models, discrete event simulation, or system dynamics, could offer a better framework for representing the temporal complexity and adaptive nature of machine learning-based clinical tools [40,41,42].

## 5. Conclusions

This study provides an early economic evaluation of a machine learning-based predictor to guide decision-making regarding resilience-strengthening psychosocial support in breast cancer treatment. The results suggest potential value in clinician QoL prediction with the aid of the QoL predictor.

## Figures and Tables

**Table 1:** Sensitivity and specificity of the QoL prediction strategies derived from the Nuutinen et al. 2023 user experiment [28]. QoL: quality of life.

**Clinician-only prediction (Strategy i)**	
Sensitivity	0.705
Specificity	0.733
**Clinician prediction supported by the QoL predictor (Strategy ii)**	
Sensitivity	0.733
Specificity	0.745
**QoL predictor alone (Strategy iii)**	
Sensitivity	0.585
Specificity	0.776

**Table 2:** Model parameters and uncertainty distributions. QoL: quality of life.

	Base Case Value	Probabilistic Sensitivity Analysis Distribution	Base Case Source
**Patient variables**			
Average QoL of patient with low QoL	0.63	Not varied	Pettini G et al. 2022 [29]
Average QoL of patient with high QoL	0.85	Not varied	Pettini G et al. 2022 [29]
Healthcare service use low QoL, 12-month period	19 visits	Not varied	Pettini G et al. 2022 [29]
Healthcare service use high QoL, 12-month period	15 visits	Not varied	Pettini G et al. 2022 [29]
Sick leave days low QoL, 12-month period	130 days	Not varied	Leskelä et al. 2023 [32]
Sick leave days high QoL, 12-month period	86 days	Not varied	Leskelä et al. 2023 [32]
Healthcare service use coefficient (how much an increase of 0.01 points in QoL reduces healthcare service use)	0.2 visits less	Normal	Pettini G et al. 2022 [29]
Sick leave coefficient (how much an increase of 0.01 points in QoL reduces sick leave)	1.2 days less	Normal	Leskelä et al. 2023 [32]
Health outcomes			
Individual psychosocial support	+0.08 QoL	Beta	Arving C et al. 2007 [30]
Costs (EUR)			
Individual psychosocial support cost for patient (5 visits)	EUR 64	Gamma	Mäklin and Kokko 2021 [34]
Individual psychosocial support cost for provider (5 visits)	EUR 947	Gamma	Mäklin and Kokko 2021 [34]
Healthcare resource use cost for patient	EUR 23 per visit	Gamma	Mäklin and Kokko 2021 [34]
Healthcare resource use cost for provider	EUR 344 per visit	Gamma	Mäklin and Kokko 2021 [34]
Sick leave day cost	EUR 378	Not varied	Confederation of Finnish industries [35]
Predicting, clinician alone	EUR 30 per prediction	Gamma	Mäklin and Kokko 2021 [34], expert opinion
Predicting, clinician with the aid of the QoL predictor	EUR 15 per prediction	Gamma	Mäklin and Kokko 2021 [34], expert opinion
Predicting, QoL predictor alone	EUR 10 per prediction	Gamma	Assumption

**Table 3:** Incremental cost-effectiveness analysis of the four decision-making strategies. NMB, net monetary benefit; QALYs, quality-adjusted life years; ICER, incremental cost-effectiveness ratio; Δ, incremental difference compared with the previous strategy; QoL, quality of life.

	NMB	Cost	Δ Cost vs. Previous	QALYs	Δ QALYs vs. Previous	ICER vs. Previous
Strategy iv (no QoL prediction, no psychosocial support)	16,252	6104	-	0.745	-	Reference
Strategy iii (QoL predictor only)	16,327	6354	+250	0.756	+0.011	Extendedly dominated
Strategy ii (clinician with QoL predictor)	16,348	6414	+60	0.759	+0.003	22,892
Strategy i (clinician only)	16,327	6420	+6	0.758	−0.001	Dominated

## Data Availability

The original contributions presented in this study are included in the article. Further inquiries can be directed to the corresponding author.

## References

[1] European Cancer Information System (2023). Breast Cancer Burden in EU-24. https://ecis.jrc.ec.europa.eu.

[2] Lam W.W.T., Shing Y.T., Bonanno G.A., Mancini A.D., Fielding R. (2012). Distress trajectories at the first year diagnosis of breast cancer in relation to 6 years survivorship. Psychooncology.

[3] Bonanno G.A., Westphal M., Mancini A.D. (2011). Resilience to loss and potential trauma. Annu. Rev. Clin. Psychol..

[4] Eicher M., Matzka M., Dubey C., White K. (2015). Resilience in adult cancer care: An integrative literature review. Oncol. Nurs. Forum.

[5] Ye Z.J., Qiu H.Z., Li P.F., Liang M.Z., Zhu Y.F., Zeng Z., Hu G.Y., Wang S.N., Quan X.M. (2017). Predicting changes in quality of life and emotional distress in Chinese patients with lung, gastric, and colon-rectal cancer diagnoses: The role of psychological resilience. Psychooncology.

[6] Duan-Porter W., Cohen H.J., Demark-Wahnefried W., Sloane R., Pendergast J.F., Snyder D.C., Morey M.C. (2016). Physical resilience of older cancer survivors: An emerging concept. J. Geriatr. Oncol..

[7] Matzka M., Mayer H., Köck-Hódi S., Moses-Passini C., Dubey C., Jahn P., Schneeweiss S., Eicher M. (2016). Relationship between Resilience, Psychological Distress and Physical Activity in Cancer Patients: A Cross-Sectional Observation Study. PLoS ONE.

[8] Popa-Velea O., Diaconescu L., Jidveian Popescu M., Truţescu C. (2017). Resilience and active coping style: Effects on the self-reported quality of life in cancer patients. Int. J. Psychiatry Med..

[9] Schumacher A., Sauerland C., Silling G., Berdel W.E., Stelljes M. (2014). Resilience in patients after allogeneic stem cell transplantation. Support Care Cancer.

[10] Wenzel L.B., Donnelly J.P., Fowler J.M., Habbal R., Taylor T.H., Aziz N., Cella D. (2002). Resilience, reflection, and residual stress in ovarian cancer survivorship: A gynecologic oncology group study. Psychooncology.

[11] Somasundaram R.O., Devamani K.A. (2016). A Comparative Study on Resilience, Perceived Social Support and Hopelessness Among Cancer Patients Treated with Curative and Palliative Care. Indian J. Palliat. Care.

[12] Zhu J., Ebert L., Wai-Chi Chan S. (2017). Integrative Review on the Effectiveness of Internet-Based Interactive Programs for Women With Breast Cancer Undergoing Treatment. Oncol. Nurs. Forum.

[13] Lipsett A., Barrett S., Haruna F., Mustian K., O’Donovan A. (2017). The impact of exercise during adjuvant radiotherapy for breast cancer on fatigue and quality of life: A systematic review and meta-analysis. Breast.

[14] Vehmanen L., Mattson J., Karademas E., Oliveira-Maia A.J., Sousa B., Pat-Horenczyk R., Mazzocco K., Simos P., Cardoso F., Pettini G. (2022). Associations between Physical Exercise, Quality of Life, Psychological Symptoms and Treatment Side Effects in Early Breast Cancer. Breast J..

[15] Pat-Horenczyk R., Kelada L., Kolokotroni E., Stamatakos G., Dahabre R., Bentley G., Perry S., Karademas E.C., Simos P., Poikonen-Saksela P. (2023). Trajectories of Quality of Life among an International Sample of Women during the First Year after the Diagnosis of Early Breast Cancer: A Latent Growth Curve Analysis. Cancers.

[16] Seiler A., Jenewein J. (2019). Resilience in Cancer Patients. Front. Psychiatry.

[17] Sutton R.T., Pincock D., Baumgart D.C., Sadowski D.C., Fedorak R.N., Kroeker K.I. (2020). An overview of clinical decision support systems: Benefits, risks, and strategies for success. NPJ Digit. Med..

[18] Jang S., Song H., Shin Y.J., Kim J., Kim J., Lee K.W., Lee S.S., Lee W., Lee S., Lee K.H. (2020). Deep Learning-based Automatic Detection Algorithm for Reducing Overlooked Lung Cancers on Chest Radiographs. Radiology.

[19] Kozuka T., Matsukubo Y., Kadoba T., Oda T., Suzuki A., Hyodo T., Im S., Kaida H., Yagyu Y., Tsurusaki M. (2020). Efficiency of a computer-aided diagnosis (CAD) system with deep learning in detection of pulmonary nodules on 1-mm-thick images of computed tomography. Jpn. J. Radiol..

[20] Sim Y., Chung M.J., Kotter E., Yune S., Kim M., Do S., Han K., Kim H., Yang S., Lee D.J. (2020). Deep Convolutional Neural Network-based Software Improves Radiologist Detection of Malignant Lung Nodules on Chest Radiographs. Radiology.

[21] Steiner D.F., MacDonald R., Liu Y., Truszkowski P., Hipp J.D., Gammage C., Thng F., Peng L., Stumpe M.C. (2018). Impact of Deep Learning Assistance on the Histopathologic Review of Lymph Nodes for Metastatic Breast Cancer. Am. J. Surg. Pathol..

[22] Kiani A., Uyumazturk B., Rajpurkar P., Wang A., Gao R., Jones E., Yu Y., Langlotz C.P., Ball R.L., Montine T.J. (2020). Impact of a deep learning assistant on the histopathologic classification of liver cancer. npj Digit. Med..

[23] Cha K.H., Hadjiiski L.M., Cohan R.H., Chan H.P., Caoili E.M., Davenport M.S., Samala R.K., Weizer A.Z., Alva A., Kirova-Nedyalkova G. (2019). Diagnostic Accuracy of CT for Prediction of Bladder Cancer Treatment Response with and without Computerized Decision Support. Acad. Radiol..

[24] Hendrix N., Veenstra D.L., Cheng M., Anderson N.C., Verguet S. (2022). Assessing the Economic Value of Clinical Artificial Intelligence: Challenges and Opportunities. Value Health.

[25] Friedman C., Wyatt J., Ash J. (2022). Evaluation Methods in Biomedical and Health Informatics.

[26] Hakkarainen T., Ira H., Riikka-Leena L. (2025). Methodological approaches in the economic evaluation of prognostic and predictive companion diagnostics: A systematic scoping review. Expert Rev. Pharmacoeconomics Outcomes Res..

[27] Kourou K., Manikis G., Mylona E., Poikonen-Saksela P., Mazzocco K., Pat-Horenczyk R., Sousa B., Oliveira-Maia A.J., Mattson J., Roziner I. (2023). Personalized prediction of one-year mental health deterioration using adaptive learning algorithms: A multicenter breast cancer prospective study. Sci. Rep..

[28] Nuutinen M., Hiltunen A.M., Korhonen S., Haavisto I., Poikonen-Saksela P., Mattson J., Manikis G., Kondylakis H., Simos P., Mazzocco K. (2023). Aid of a machine learning algorithm can improve clinician predictions of patient quality of life during breast cancer treatments. Health Technol..

[29] Pettini G., Sanchini V., Pat-Horenczyk R., Sousa B., Masiero M., Marzorati C., Galimberti V.E., Munzone E., Mattson J., Vehmanen L. (2022). Predicting Effective Adaptation to Breast Cancer to Help Women BOUNCE Back: Protocol for a Multicenter Clinical Pilot Study. JMIR Res. Protoc..

[30] Arving C., Sjode P.O., Bergh J., Hellbom M., Johansson B., Glimelius B., Brandberg Y. (2007). Individual psychosocial support for breast cancer patients: A randomized study of nurse versus psychologist interventions and standard care. Cancer Nurs..

[31] Aaronson N.K., Ahmedzai S., Bergman B., Bullinger M., Cull A., Duez N.J., Filiberti A., Flechtner H., Fleishman S.B., Haes J.C.D. (1993). The European-Organization-For-Research-And-Treatment-Of-Cancer QLQ-C30—A Quality-Of-Life Instrument for Use in International Clinical-Trials in Oncology. J. Natl. Cancer Inst..

[32] Leskelä R.L., Haavisto I., Pennanen P., Lahelma M., Mattson J., Poikonen-Saksela P. (2023). Predictive factors for prolonged sick leave in breast cancer patients treated with adjuvant therapies: A retrospective registry study. Acta Oncol..

[33] Kapiainen S., Väisänen A., Haula T. Terveyden- ja Sosiaalihuollon Yksikkökustannukset Suomessa Vuonna 2011. https://www.google.com/url?sa=t&source=web&rct=j&opi=89978449&url=https://core.ac.uk/download/pdf/19529454.pdf&ved=2ahUKEwif9rfWm6iSAxUeTGwGHV1sJd4QFnoECBwQAQ&usg=AOvVaw2w-i2mkc5AsV9I3tZpTsrZ.

[34] Mäklin S., Kokko P. (2021). Terveyden- ja Sosiaalihuollon Yksikkökustannukset Suomessa Vuonna 2017. https://www.julkari.fi/handle/10024/142882.

[35] Confederation of Finnish Industries Cost of Sick Leave Day for Employer. https://ek.fi/ajankohtaista/uutiset/paivan-sairauspoissaolosta-aiheutuu-tyonantajalle-keskimaarin-370-euron-kustannus/.

[36] (2025). Producer Price Indices for Services | Statistics Finland. https://stat.fi/en/statistics/pthi.

[37] Gelman A., Carlin J.B., Stern H.S., Rubin D.B. (2014). Bayesian Data Analysis.

[38] Husereau D., Drummond M., Petrou S., Carswell C., Moher D., Greenberg D., Augustovski F., Briggs A.H., Mauskopf J., Loder E. (2013). Consolidated Health Economic Evaluation Reporting Standards (CHEERS)—Explanation and Elaboration: A Report of the ISPOR Health Economic Evaluation Publication Guidelines Good Reporting Practices Task Force. Value Health.

[39] Vellekoop H., Huygens S., Versteegh M., Szilberhorn L., Zelei T., Nagy B., Koleva-Kolarova R., Tsiachristas A., Wordsworth S., Rutten-van Mölken M. (2021). Guidance for the Harmonisation and Improvement of Economic Evaluations of Personalised Medicine. Pharmacoeconomics.

[40] Marshall D.A., Grazziotin L.R., Regier D.A., Wordsworth S., Buchanan J., Phillips K., Ijzerman M. (2020). Addressing Challenges of Economic Evaluation in Precision Medicine Using Dynamic Simulation Modeling. Value Health.

[41] Marshall D.A., Burgos-Liz L., IJzerman M.J., Osgood N.D., Padula W.V., Higashi M.K., Wong P.K., Pasupathy K.S., Crown W. (2015). Applying Dynamic Simulation Modeling Methods in Health Care Delivery Research—The SIMULATE Checklist: Report of the ISPOR Simulation Modeling Emerging Good Practices Task Force. Value Health.

[42] Marshall D.A., Burgos-Liz L., IJzerman M.J., Crown W., Padula W.V., Wong P.K., Pasupathy K.S., Higashi M.K., Osgood N.D. (2015). Selecting a dynamic simulation modeling method for health care delivery research-part 2: Report of the ISPOR Dynamic Simulation Modeling Emerging Good Practices Task Force. Value Health.

